# Removal and bypass of restriction modification systems increases transformation efficiency of *Thermococcus kodakarensis*

**DOI:** 10.3389/fmicb.2026.1855967

**Published:** 2026-07-07

**Authors:** Alexander M. Alon, Christopher M. Sanders, Brett W. Burkhart, Thomas J. Santangelo

**Affiliations:** 1 Department of Biochemistry and Molecular Biology, Colorado State University, Fort Collins, CO, United States; 2 Graduate Program in Cell and Molecular Biology, Colorado State University, Fort Collins, CO, United States

**Keywords:** archaea, genetics, hyperthermophile, McrBC, restriction-modification systems, transformation

## Abstract

Archaea are increasingly recognized as key participants in global nutrient cycles, as idealized platforms for biotechnological applications, and as the progenitors of Eukarya. Despite such importance, limited genetic access to most archaeal taxa limits validation of key hypotheses, mandating a better understanding of archaeal transformation mechanisms and archaeal-encoding host-defense systems that typically hinder genetic competence. The hyperthermophilic marine species *Thermococcus kodakarensis* (*T. kodakarensis)* has emerged as a premier model archaeal species in large part due to natural competency and a wealth of genetic tools to manipulate the genome or ectopically complement strains with autonomously replicating plasmids. Despite continued successes, a limited number of genetic selections can be applied to specific type-strains of *T. kodakarensis*. Known restriction modification (RM) systems and other unknown host defense systems present in all strain variants reduce overall transformation efficiencies. We report here a unified *T. kodakarensis* strain (TS900) with a genotype that combines all the selective and counter-selective pressures currently available, that is compatible with all existing genetic procedures and plasmids.TS900-derived strains genetically deleted for two known Type II RM systems and a pair of McrBC endonucleases demonstrate dramatically enhanced transformation efficiency, opening *T. kodakarensis* to new approaches that demand efficient DNA uptake and genomic integration. Additionally, transformations with unmodified plasmid DNA likely bypasses host RM systems, further improving transformation efficiency. The unified genetic background, massively increased competence, and use of unmodified plasmid DNA in transformations will open new arenas of archaeal biology and the investigation of life in the extremes for experimental study.

## Introduction

Studies of archaeal biology have provided a wealth of information regarding physiology in the extremes (Sato et al., 2007; Sorokin et al., 2017; Burkhart et al., 2019), evolutionary connections of archaeal information processing systems with bacteria and eukaryotes (Hirata et al., 2008; Cubonová et al., 2013; Spang et al., 2015; Zaremba-Niedzwiedzka et al., 2017), and detailed avenues to exploit archaeal biology for profitable biotechnology applications (Atomi et al., 2011; Yin et al., 2015; Dumorné et al., 2017; Quehenberger et al., 2017; Singh and Singh, 2017; Cabrera and Blamey, 2018; Enzmann et al., 2018; Straub et al., 2018; Schocke et al., 2019; De Lise et al., 2023). Many experimental gains in the Archaea have emerged from a relatively small number of model species that share the common feature of genetic accessibility (Berkner and Lipps, 2008; Leigh et al., 2011; Atomi et al., 2012; Hileman and Santangelo, 2012; Farkas et al., 2013; Gehring et al., 2017; Peng et al., 2017; Liman et al., 2022). Genome engineering to alter gene expression, delete or introduce new genes and activities, combined with introduction of replicative expression plasmids provides a powerful set of tools to investigate archaeal biology.

Genetic systems to manipulate select halophilic- and methanogenic-archaea are perhaps the oldest, and in many ways provide the greatest flexibility to alter physiology and gene expression (Bertani and Baresi, 1987; Charlebois et al., 1987; Cline and Doolittle, 1987; Tumbula et al., 1994; Metcalf et al., 1997; Rother and Metcalf, 2005; Soppa, 2006; Guss et al., 2008; Kohler and Metcalf, 2012; Farkas et al., 2013; Fink et al., 2021, 2023). With the exception of the recently developed genetic system for *Methanocaldococcus jannaschii* (Susanti et al., 2019), however, these often-efficient methanogen and halophilic genetic systems do not permit study of hyperthermophilic biology. Experimental manipulation of several members of the Sulfolobales (Mao and Grogan, 2017; Peng et al., 2017; Schocke et al., 2019), *S. solfataricus* (Kurosawa and Grogan, 2005; Wagner et al., 2012), *S. acidocaldarius* (Schleper et al., 1992; Berkner and Lipps, 2008), and *S. islandicus* (Deng et al., 2009; She et al., 2009; Zhang and Whitaker, 2012, 2017; Zhang et al., 2013, 2016, 2018), has provided insight into single-gene and genome-wide requirements for activities supporting life of these aerobic, TACK-clade model hyperthermophiles. Investigation of the remaining hyperthermophilic archaeal species has primarily employed members of the Thermococcales (Sato et al., 2003, 2005; Prieur et al., 2004; Matsumi et al., 2007; Lipscomb et al., 2011; Farkas et al., 2012; Hileman and Santangelo, 2012; Gehring et al., 2017; Song et al., 2021; Liman et al., 2022; Catchpole et al., 2023), including *Pyrococcus furiosus* (Waege et al., 2010; Kreuzer et al., 2013), a super-competent derivative of *P. furiosus* termed COM1 (Lipscomb et al., 2011; Bridger et al., 2012; Farkas et al., 2012), *Pyrococcus yayanosii* (Li et al., 2015; Thiel et al., 2014; Song et al., 2019, 2021), *Thermococcus onnurineus NA1* (Kim et al., 2013; Lee et al., 2015), *Thermococcus barophilus* (Thiel et al., 2014; Birien et al., 2018) and *Thermococcus kodakarensis* (originally termed *Pyrococcus kodakaraensis-KOD1* (Morikawa et al., 1994; Fujiwara et al., 1998), then *Thermococcus kodakaraensis-KOD1* (Sato et al., 2003, 2005; Atomi et al., 2004; Matsumi et al., 2007; Hileman and Santangelo, 2012; Farkas et al., 2013; Gehring et al., 2017; Song et al., 2020, 2021; Liman et al., 2022). The genetic systems originally derived for *T. kodakarensis* (Sato et al., 2003) form the basis for essentially all of the techniques employed in the evolutionary-close fermentative Thermococcal species that thrive in marine hyperthermophilic environments.

Genetic manipulations of *T. kodakarensis* are challenged both by identification of tight selective markers that function in the extremes of temperature and overcoming host-defense systems. Most archaeal genomes, including *T. kodakarensis*, employ both CRISPR defense systems and more classically described restriction modification (RM) systems (Zatopek et al., 2021). While the former can often be manipulated to aid in genomic transformations, the endonuclease activities of RM systems are almost universally disruptive of transformations. The most common RM systems - Type IIP – encode distinct enzymes that cleave or methylate palindromic sequences, respectively, while Type IIG (single polypeptide systems that both methylate and cleave recognition sequences) and Type IIL RM systems (again single enzyme systems that only hemi-methylate or cleave asymmetric sites) have also been identified in several archaeal clades. Removal of the two Type IIL enzymes (TkoI and TkoII) significantly increases transformation efficiency of *T. kodakarensis*, but overall transformation efficiencies remain relatively low, suggesting that elimination of additional systems may further improve transformation efficiencies.

In contrast to Type II RM systems, Type IV RM systems are modification-dependent restriction systems, targeting foreign DNAs with existing modifications that are not typically present on the host genome. Two component McrBC systems-so named for modified cytosine restriction-are perhaps the best described Type IV RM systems, with a dependency on either methyl-5-cytosine (m^5^C) or 5-hydroxymethyl-cytosine (5hmC) recognition on invader DNA to initiate cleavage. McrB is, perhaps confusingly, a GTP- rather than ATP-dependent AAA+ family protein that binds the McrC nuclease. McrB must recognize two m^5^C or 5hmC sites to initiate McrC cleavage near, but not at the modified cytosine residues (Raleigh, 1992; Sutherland et al., 1992).

We removed all bioinformatically identified RM systems (Type II and Type IV) within a genetically unified strain to accelerate future progress in strain development (Chung et al., 2013; Johnston et al., 2019; Zhang et al., 2020; Finn et al., 2021). We first generated a strain (TS900) that combines all currently available genetic selections for *T. kodakarensis*. To further enhance the genetic prowess of *T. kodakarensis* we individually, then in combination, deleted both Type II and both Type IV RM systems within this strain background, resulting in strains with transformation efficiencies as high as 1 in 1,000 cells, a three to four order of magnitude increase in transformation efficiency. The development of a universal genomic platform compatible with all genetic selections and autonomously replicating plasmids for *T. kodakarensis* and massively increased transformation efficiency provides avenues for increased genetic investigation of the hyperthermophilic Thermococcales.

## Material and methods

### Microbial growth and media conditions

*T. kodakarensis* strains were grown as previously described (Hileman and Santangelo, 2012; Gehring et al., 2017; Zatopek et al., 2021; Liman et al., 2022) in artificial sea water (ASW) medium supplemented with vitamins and trace minerals. 5 g/l yeast extract (Y), 5 g/l tryptone (T), 5 g/l pyruvate (Pyr) and 2 g/l elemental sulfur (S°) were added to nutrient-rich medium (ASW-YT-Pyr-S°). An ASW-S° mixture supplemented with a combination of 19 (lacking tryptophan) or 20 amino acids formed minimal medium (ASW-aa-S°). All cultures were grown under strict anaerobic conditions at 85 °C. When necessary, cultures were supplemented with 1 mM agmatine. Solid medium was prepared by the addition of 1% gelzan, with polysulfides substituting for S° (Santangelo et al., 2007, 2010). Growth of liquid cultures was monitored by measurements of optical density at 600 nm in biological quadruplicate.

### Construction of *T. kodakarensis* strain TS900

Plasmids used to direct the markerless-deletion of genomic sequences were each individually constructed from the parental plasmid pTS700 (Gehring et al., 2017; Liman et al., 2022) and contain ~500-700 bp sequences complementary to both upstream and downstream regions of the respectively locus under study (Gehring et al., 2017; Liman et al., 2022). Each vector also encodes expression cassettes for TK0149 (provides agmatine autotrophy) and TK0664 (provides sensitivity to 6-MP). Strains were constructed as previously described (Fukuda et al., 2008; Santangelo et al., 2010; Hileman and Santangelo, 2012; Farkas et al., 2013; Gehring et al., 2017; Liman et al., 2022). Briefly, plasmids incapable of autonomous replication in *T. kodakarensis* were individually transformed into *T. kodakarensis* TS559 (ΔTK0149; ΔTK0664; ΔTK0254::TK2276; ΔTK2276) (Santangelo et al., 2010; Hileman and Santangelo, 2012; Farkas et al., 2013; Gehring et al., 2017; Liman et al., 2022). Plasmid integration at the desired locus was confirmed by several diagnostic PCR amplicons generated from genomic DNA purified from intermediate strains. Overnight growth in the presence of 1 mM agmatine permitted spontaneous plasmid excision, and colonies were selected on solid media containing 20 amino acids, 6-methyl purine (6-MP) and agmatine. DNA was extracted from 1 ml ASW-YT-Pyr-S°-agmatine cultures grown from individual 6-MP resistant colonies for use in diagnostic PCRs to confirm deletion/modification of the desired locus.

### Construction of *T. kodakarensis* strain TS901

Plasmids used to direct the markerless-deletion of genomic sequences encoding TK1158 and TK1460 were sequentially employed, as previously described (Gehring et al., 2017; Liman et al., 2022), in strain TS900 to ultimately generate TS901. Competency was determined as described previously (Gehring et al., 2017; Liman et al., 2022).

### Construction of *T. kodakarensis* strains TS902, TS903, and TS904

A plasmid was used to direct the markerless-deletion of genomic sequences encoding TK0794 and TK0795 was employed, as previously described (Fukuda et al., 2008; Santangelo et al., 2010; Hileman and Santangelo, 2012; Farkas et al., 2013; Gehring et al., 2017; Liman et al., 2022), in strain TS901 to ultimately generate TS902. A plasmid was used to direct the markerless-deletion of genomic sequences encoding TK1009 and TK1010 was employed, as previously described (Fukuda et al., 2008; Santangelo et al., 2010; Hileman and Santangelo, 2012; Farkas et al., 2013; Gehring et al., 2017; Liman et al., 2022), in strain TS901 to ultimately generate TS903. Finally, the plasmid used to direct the markerless-deletion of genomic sequences encoding TK1009 and TK1010 was employed, as previously described (Fukuda et al., 2008; Santangelo et al., 2010; Hileman and Santangelo, 2012; Farkas et al., 2013; Gehring et al., 2017; Liman et al., 2022), in strain TS902 to ultimately generate TS904. Competency was determined as described previously (Zatopek et al., 2021). Markerless deletion was confirmed by PCR screening and whole-genome sequencing.

### DNA preparations and PCR

Preparations of genomic DNA from cultures of *T. kodakarensis* were purified as previously described (Santangelo et al., 2007). Primer sequences for diagnostic PCRs to confirm genotypes are listed in Supplementary materials.

#### Modified deoxyribonucleoside detection

For modified deoxyribonucleoside detection, genomic DNAs from strains TS559, TS900, TS901, TS902, TS903, and TS904 were digested to nucleosides at 37 °C overnight using a Nucleoside Digestion Mix (NEB, Cat #M0649S). The digested DNAs were subsequently injected without prior purification on an Agilent 1,290 Infinity II UHPLC equipped with a G7117 diode array detector and an Agilent 6495C Triple-Quadrupole Mass Spectrometer operating in positive electrospray ionization (+ESI) mode. UHPLC was conducted on a Waters X select HSS T3 XP column (2.1 × 100 mm, 2.5 μm) containing methanol and 10 mM ammonium acetate (pH 4.5) gradient mobile phase. Mass spectrometric data were acquired using dynamic multiple reaction monitoring (DMRM) mode. Identification of each deoxyribonucleoside species was based on the associated retention time and mass transition (e.g., dA: 252.1 -> 136.1; dm6A: 266.1 -> 150.1) in the extracted ion chromatogram.

### Generation of methylated and unmethylated plasmid DNA for transformation efficiency assays

pTS543 (Santangelo et al., 2010) and non-replicative, pTS700-derived plasmid (p1827SurRsite) were transformed into Stellar *E. coli* (HST08 strain) or *dam*^−^*/dcm*^−^ *E. coli* (New England Biolabs, Inc. Catalog # C2925H) and plated on LB containing 100 μg/mL ampicillin. 100 mL cultures were used for plasmid extraction using a Qiagen Plasmid Midi Purification Kit (Catalog# 12143). The methylation status of plasmids extracted from either *E. coli* strain was confirmed by incubation with DpnI or MspJI. The presence of unidentified RM systems was confirmed by incubating extracted plasmid DNAs with TS559 or TS904 cell lysates for 30 min at 85 °C before resolving DNA by gel electrophoresis.

### Transformation efficiency assays

Transformations demanding either retention of autonomously replicating plasmids or plasmids that must integrate into the genome were completed with strains TS559, TS900, TS901, TS902, TS903, and TS904 as previously described (Gehring et al., 2017; Liman et al., 2022), employing identical plasmids recovered from *dam*^+^*/dcm*^+^ or *dam*^−^*/dcm*^−^ *E. coli*. Suspensions of the transformed strain were spotted in 1:10 serial dilutions on rich-media plates lacking agmatine and were incubated at 85 °C for 60 h. Colonies formed on solid medium were observed by lifting cells to PVDF membranes before staining with Coomassie brilliant blue. Colonies from the most diluted spot were back calculated to represent the number of transformed or parental colonies in the reaction volume. Transformation efficiency was measured as the number of transformants / colony forming units. Assays were completed in minimally three replicates.

## Results

### The genotype of strain TS900 permits the combinatorial use of all extant procedures for modification of the *T. kodakarensis* genome

Two selective and counter-selective genetic techniques are primarily used to generate genomic modifications in *T. kodakarensis* (Sato et al., 2003, 2005; Santangelo et al., 2010; Hileman and Santangelo, 2012; Farkas et al., 2013; Gehring et al., 2017; Liman et al., 2022). The first is reliant on selection for uracil prototrophy upon transformation of an uracil auxotrophic strain; strain KU216 serves as the primary host for uracil-based selections (Sato et al., 2005). Integration of the exogenous DNA into the KU216 genome results in uracil prototrophy but also sensitivity to 5-fluororatic acid (5-FOA) due to the activity of orotidine-5′-phosphate decarboxylase encoded by *pyrF* (TK2276) on the exogenous DNA (Sato et al., 2003). Spontaneous excision of most of the exogenous DNA that integrated into the KU216 genome through recombination between directly repeated sequences that were generated in the genome during the initial integration event is sufficiently frequent to select for cells that have reverted to uracil auxotrophy and become 5-FOA resistant. This single-gene selective and counter-selective system permits rapid strain construction and is only commonly complicated by contaminating amounts of uracil in some components of *T. kodakarensis* growth media (Farkas et al., 2013). The only requirements of the host strain is that the genome initially lacks *pyrF* activity, and the KU216 genome has a complete deletion of TK2276 (Sato et al., 2003).

The second commonly employed genetic procedure for generating markerless modifications of the *T. kodakarensis* genome is reliant on a two-gene selection and counter-selection procedure; strain TS559 serves as the primary host for such procedures (Santangelo et al., 2010; Hileman and Santangelo, 2012; Gehring et al., 2017; Liman et al., 2022). Initial transformants of strain TS559 are selected based on incorporation of exogenous DNA that either restores agmatine prototrophy through expression of pyruvoyl-dependent arginine-decarboxylase (*pdaD*; TK0149) (Fukuda et al., 2008; Santangelo et al., 2010; Gehring et al., 2017; Liman et al., 2022) on nutrient rich-medium lacking agmatine or through restoration of tryptophan prototrophy through expression of anthranilate synthase I (*trpE*; TK0254) (Sato et al., 2003; Hileman and Santangelo, 2012) on nutrient-poor, 19 amino-acid based minimal medium lacking tryptophan. While both selection pressures are tight and have no recorded background, the agmatine-based selection is more often employed as it yields transformants faster as agmatine prototrophy can be selected on nutrient rich-medium. Integration of either the *trpE* or *pdaD* expression cassette necessarily co-integrates a second expression cassette for a hypoxanthine-guanine phosphoribosyl-transferase (HGPRT) encoded by TK0664 (Santangelo et al., 2010; Gehring et al., 2017; Liman et al., 2022). HGPRT activity converts TS559 from a strain initially resistant to the cytotoxic properties of 6-methyl purine (6-MP) to a strain sensitive to 6-MP. The genotype of the host is more constrained for this second, two-gene selective and counter-selective strategy and demands the deletion of TK0149 and/or TK0254 for selections, and deletion of TK0664 for counter-selective pressures; the genotype of TS559 (Santangelo et al., 2010; Gehring et al., 2017; Liman et al., 2022) (ΔTK2276 (*pyrF);* ΔTK0254 (*trpE*)::TK2276; ΔTK0664 (HGPRT); ΔTK0149 (*pdaD*)) is properly tailored for such two-gene selective pressures.

**Figure 1:**
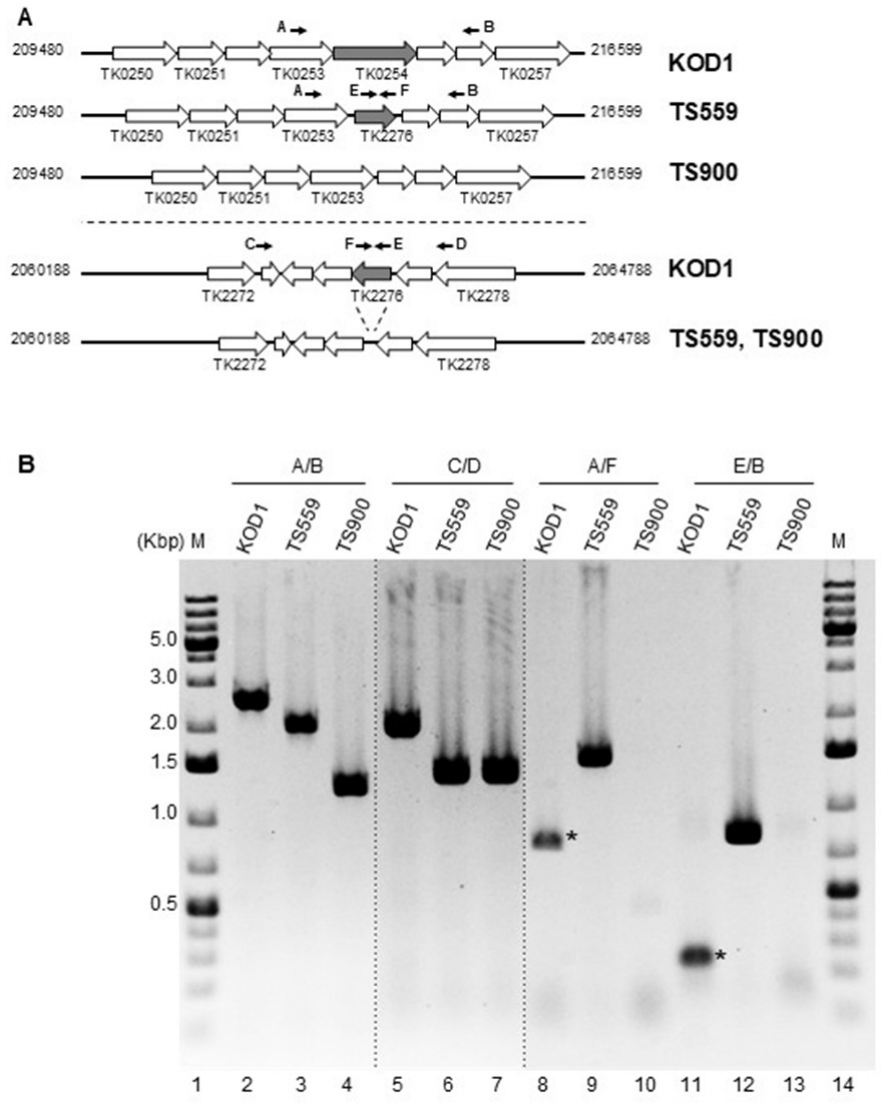
The TS900 genome supports use of all existing genetic selections and counterselections for *T. kodakarensis*. **(A)** Schematic map of the *T. kodakarensis* genome from strains WT-KOD1, TS559 and TS900 surrounding the TK0254 locus (top) and TK2276 locus (bottom) highlighting the binding positions of oligonucleotides (A–F) that were used in diagnostic PCRs. **(B)** PCRs with primer sets listed above each lane generate amplicons from genomic DNA purified from each *T. kodakarensis* strain. Primer pairs that flank TK0254 (A/B) generate different size amplicons for each strain, representative of retention of TK0254 in WT-KOD1 (lane 2), replacement of TK0254 by TK2276 in TS559 (lane 3), and deletion of TK0254 in TS900 (lane 4). The external primer pair (C/D) for the TK2276 locus generates a smaller amplicon from genomic DNA from TS559 and TS900 (lanes 6 and 7) reflecting loss of TK2276 coding sequences. Amplicons generated using primers complementary to TK2276 in combination with primers complementary to TK0254 flanking sequences (pairs A/E and F/B) are only generated in strains TS559 (lanes 9 and 12). *M* = DNA standards in Kbp. Stars denote aberrant, non-specific amplicons. * represents non-specific binding.

To increase the pace and flexibility of genomic modifications for *T. kodakarensis* we generated a new strain, TS900, that incorporates all the necessary genomic modifications for uracil, agmatine, tryptophan, 5-FOA, and 6-MP based selections into a single strain (Figure 1; Table 1). The genotype of TS900 (ΔTK2276 (*pyrF);* ΔTK0254 (*trpE*); ΔTK0664 (HGPRT); ΔTK0149 (*pdaD*)) permits use of both common genetic strategies for markerless modification of the *T. kodakarensis* genome. TS900 is derived from TS559, but whereas TS559 retains a scarred genome through inactivating TK0254 by insertion of TK2276 (ΔTK0254::TK2276). The genome of TS900 is markerlessly and cleanly deleted for TK2276 at the original locus (ΔTK2276 (*pyrF)*), and also cleanly deletes TK2276 from the TK0254 locus, resulting in a markerless deletion of TK0254. PCR amplicons from WT-KOD1, TS559, and TS900 generated with primers that flank the TK0254 (Figure 1A, upper panel) get progressively smaller as expected from the retention of TK0254 (Figure 1B, lane 3), replacement of TK0254 with the small TK2276 expression cassette (Figure 1B, lane 4), and full deletion of TK0254 sequences (Figure 1B, lane 5), respectively. Amplicons generated with primers pairs that flank the native locus of TK2276 (Figure 1A, lower panel) demonstrate the retention of TK2276 in WT-KOD1 (Figure 1B, lane 6) and clean deletion of TK2276 sequences from the native locus in strains TS559 and TS900 (Figure 1B, lanes 7 and 8). While the TK2276 expression cassette inserted at the TK0254 locus can be confirmed in strain TS559 by generation of amplicons from primer pairs that bind within TK2276 and adjacent to TK0254 (ie. pairs A/E and F/B; Figure 1B, lanes 9 and 12), such amplicons are not possible from genomic DNA of strains WT-KOD1 and TS900 that either retain TK0254 or lack TK0254 (Figure 1B, lanes 8, 10, 11, and 13; spurious amplicons are denoted with asterisks). Phenotypic analyses of TS900 are in full support of the genotype: TS900 is an uracil-, tryptophan-, and agmatine-auxotrophic strain that is sensitive to mevinolin, but resistant to 5-FOA and 6-MP (Table 1). As anticipated, the transformation efficiency of TS900 is unchanged from its direct parental strain, TS559.

**Table 1:** *T. kodakarensis* strains used and/or constructed in this study.

Ref.	Strain	Genotype	Uracil	Tryptophan	Agmatine	5-FOA	6-MP	Mevinolin
Atomi et al. (2004)	KOD1	Original isolate	Prototroph	Prototroph	Prototroph	Sensitive	Sensitive	Sensitive
Sato et al. (2005)	KU216	ΔTK2276	Auxotroph	Prototroph	Prototroph	Resistant	Sensitive	Sensitive
Sato et al. (2005)	KW128	KU216; ΔTK0254::TK2276	Prototroph	Auxotroph	Prototroph	Sensitive	Sensitive	Sensitive
Fukuda et al. (2008)	TS517	KW128; ΔTK0664	Prototroph	Auxotroph	Prototroph	Sensitive	Resistant	Sensitive
Fukuda et al. (2008)	TS559	TS517; ΔTK0149	Prototroph	Auxotroph	Auxotroph	Sensitive	Resistant	Sensitive
Zatopek et al. (2021)	ΔTkoIΔTkoII	TS559; ΔTK1158; ΔTK1460	Prototroph	Auxotroph	Auxotroph	Sensitive	Resistant	Sensitive
This study	TS900	TS559; ΔTK0254; ΔTK2276	Auxotroph	Auxotroph	Auxotroph	Resistant	Resistant	Sensitive
This study	TS901	TS900; ΔTK1158; ΔTK1460						
This study	TS902	TS901; ΔTK0794-TK0795						
This study	TS903	TS901; ΔTK1009-TK1010						
This study	TS904	TS902; ΔTK1009-TK1010						

### Removal of type IV RM systems, McrBC1 and McrBC2, impacts cellular fitness

KOD-1, and common parental strains for genetic manipulations (e.g., KU216, TS559, and now TS900) encode at least two Type IIL restriction-modification systems termed TkoI and TkoII encoded by TK1460 and TK1158, respectively (Zatopek et al., 2021). These single-protein RM systems are fused DNA methylase and endonuclease active proteins that limit transformation efficiency by often, but rarely entirely, degrading introduced DNAs designed to recombine into the genomes of KU216, TS559, or TS900. Sequential targeting TK1158, then TK1460, for markerless-deletion in strain TS900 yields strain TS901 (Table 1). TS901 is thus markerlessly deleted for six loci: TK0149, TK0254, TK0664, TK1158, TK1460, and TK2276.

**Figure 2:**
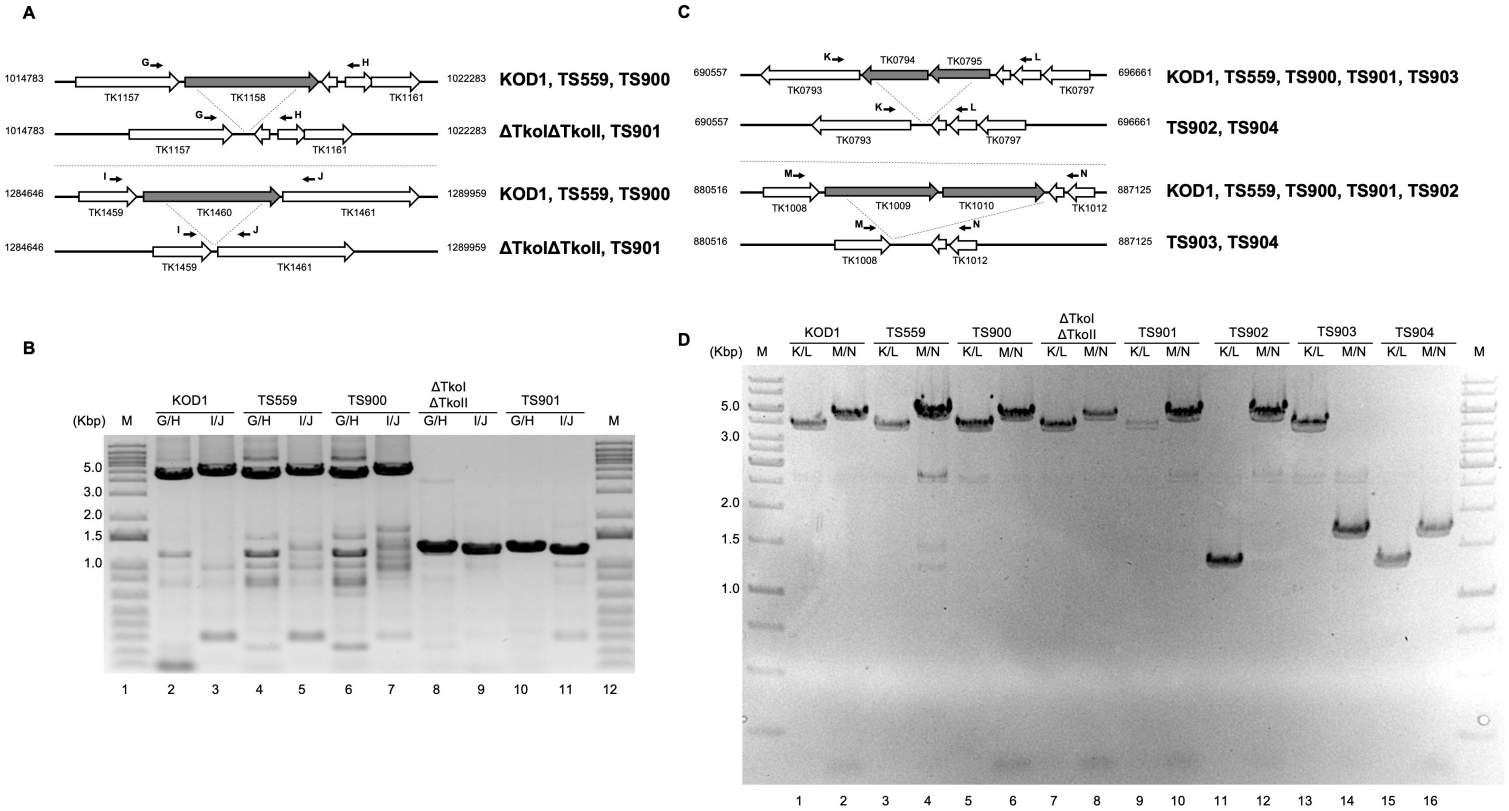
Strains TS901 – TS904 lack Type II RM systems (TkoI and TkoII) and Type IV McrBC endonuclease in a genetically unified strain **(A)** Schematic map of the *T. kodakarensis* genome from strains WT-KOD1, TS559 and TS900, TS901, and ΔTkoIΔTkoII surrounding the TK1158 locus (top) and TK1460 locus (bottom) highlighting the binding positions of oligonucleotides (G–J) that were used in diagnostic PCRs. **(B)** PCRs with primer sets listed above each lane generate amplicons from genomic DNA purified from each *T. kodakarensis* strains. Primer pairs that flank TK1158 (G/H) generate a large (~4.6 Kbp) or smaller (~1.3 Kbp) amplicon in strains retaining (KOD1, TS559, and TS900) or lacking (TS901 ΔTkoIΔTkoII) TK1158. Primer pairs that flank TK1460 (I/J) generate a large (~5.1 Kbp) or smaller (~1.3 Kbp) amplicon in strains retaining (KOD1, TS559, and TS900) or lacking (TS901 ΔTkoIΔTkoII) TK1460. **(C)** Schematic map of the *T. kodakarensis* genome from strains WT-KOD1, TS559 and TS900, TS901, ΔTkoIΔTkoII, TS902, TS903, and TS904 surrounding the TK0794-TK0795 locus (top) and TK1009-TK1010 locus (bottom) highlighting the binding positions of oligonucleotides (K-N) that were used in diagnostic PCRs. **(D)** PCRs with primer sets listed above each lane generate amplicons from genomic DNA purified from each *T. kodakarensis* strain. Primer pairs that flank TK0794 and TK0795 (K/L) generate a large (~4.8 Kbp) or smaller (~1.3 Kbp) amplicon in strains retaining (KOD1, TS559, TS900, TS901, ΔTkoIΔTkoII, and TS903) or lacking (TS902 and TS904) TK0794 and TK0795. Primer pairs that flank TK1009 and TK1010 (M/N) generate a large (~5.6 Kbp) or smaller (~1.7 Kbp) amplicon in strains retaining (KOD1, TS559, TS900, TS901, ΔTkoIΔTkoII, and TS902) or lacking (TS903 and TS904) TK1009 and TK1010.

PCR amplicons from WT-KOD1, TS559, and TS900 generated with primers that flank the TK1158 locus (Figure 2A, upper panel) demonstrate retention of TK1158 (Figure 2B, lanes 2, 4, and 6, respectively) whereas amplicons from ΔTkoI/ΔTkoII and TS901 generated with the same primer pair demonstrate loss of TK1158 from the genome (Figure 2B, lanes 8 and 10, respectively). Diagnostic PCRs to define the presence or absence of TK1460 yields similar results: PCR amplicons from WT-KOD1, TS559, and TS900 generated with primers that flank the TK1460 locus (Figure 2A, lower panel) demonstrate retention of TK1460 (Figure 2B, lanes 3, 5, and 7, respectively) whereas amplicons from ΔTkoI/ΔTkoII and TS901 generated with the same primer pair demonstrate loss of TK1460 from the genome (Figure 2B, lanes 9 and 11, respectively).

*T. kodakarensis* additionally encodes at least two homologous McrBC systems (termed McrBC1 and McrBC2), both Type IV RM enzymes that recognize 5-methylcytosines and 5-hydroxymethylcytosines (Raleigh, 1992; Sutherland et al., 1992). McrBC1 and McrBC2 are encoded as two pairs of GTPase and catalytic subunits (TK0794-TK0795 and TK1009-TK1010), respectively. We generated strains lacking TK0794-TK0795 (TS902), TK1009-TK1010 (TS903), and a strain lacking both McrBC1 and McrBC2 (TS904) using the same markerless deletion strategy (Gehring et al., 2017; Liman et al., 2022) (Table 1). Removal of McrBC components was confirmed by multiple PCRs and whole genome sequencing (WGS) (Figures 2C, D). WGS provides a mechanism to ensure newly constructed strains lack any spontaneous changes at secondary sites. To provide a platform for comparison of WGS from strains derived from TS900, TS901, TS902, TS903, and TS904 we deposited the WGS FASTA files for TS900-TS904 with GenBank at the National Center for Biotechnology Information (NCBI).

**Figure 3:**
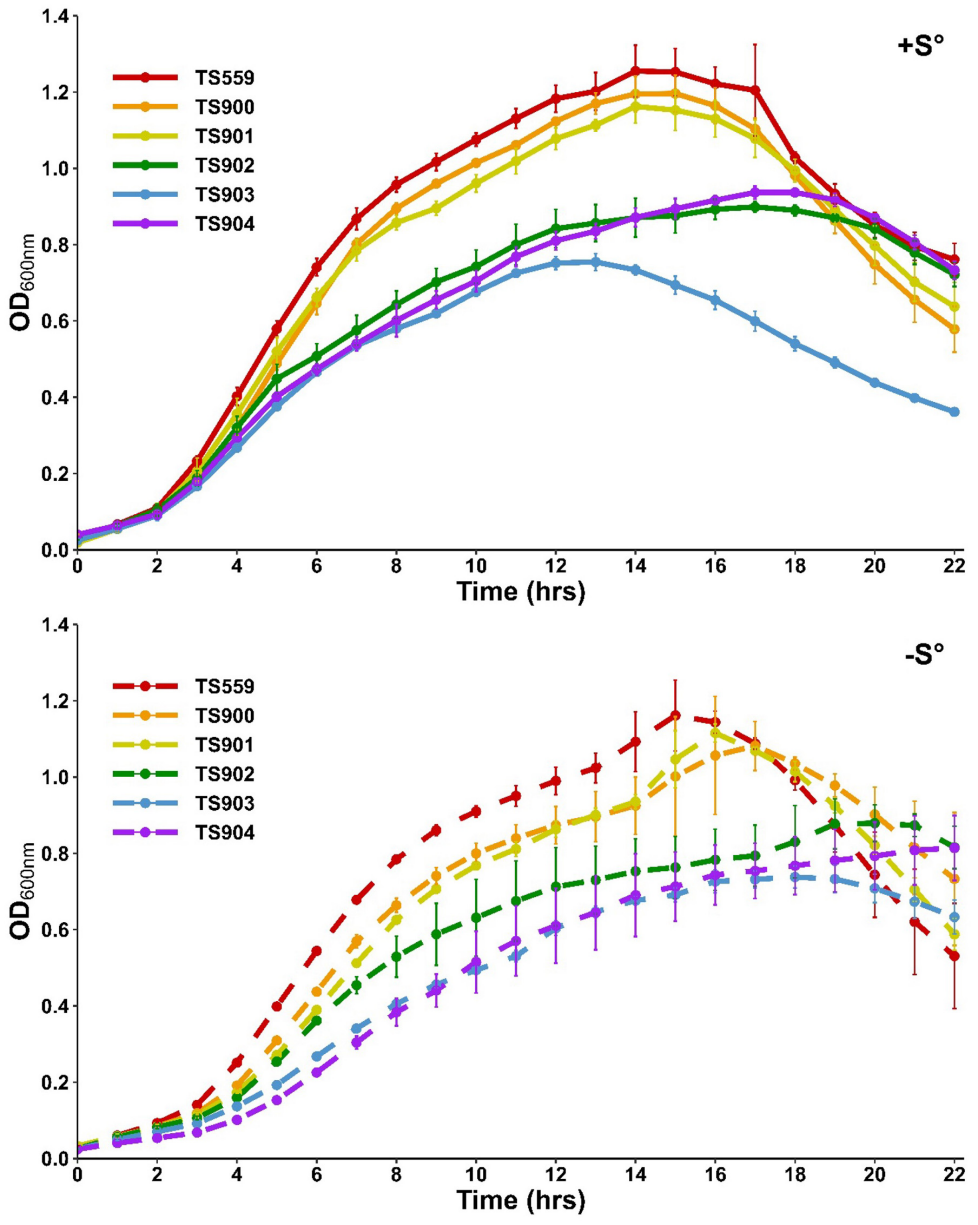
Strains lacking McrBC endonuclease components have fitness consequences. Monitoring the growth of biological quadruplicate cultures of TS559, TS900, TS901, TS902, TS903, and TS904 in the presence of absence of sulfur. The genetic changes introduced to TS599 to generate TS900, and those introduced to TS900 to generate TS901, do not impact growth rate or final cell densities. The genetic changes introduced to TS901 to generate TS902, TS903, and TS904 do impact growth rate and final cell densities. There are no disparities between the growth trends in the presence or absence of sulfur for all strains.

Whereas TS900 and TS901 grow at 85 °C in the presence or absence of sulfur at a comparable rate to TS559, strains lacking McrBC components, especially TS903, have a less pronounced exponential phase and ultimately reach lower maximum optical densities (Figure 3). While McrBC systems are known to detect and destroy modified foreign DNAs, the unanticipated impacts on overall physiology due to deletion of McrBC1 and/or McrBC2 suggest that additional roles for archaeal-encoding McrBC systems remain to be investigated.

### Removal of type IV RM McrBC systems dramatically improves transformation efficiency

We anticipated a role for both Type IIL and Type IV RM systems in restricting the efficiency of plasmid-based transformations by targeting the plasmid DNA for cleavage, resulting in plasmid loss. Our prior studies detailed the impacts of deleting both Type IIL systems (Zatopek et al., 2021), and our results here demonstrate that both Type IV systems, McrBC1 and McrBC2, play significant, but unequal roles in limiting transformation of *T. kodakarensis*. Removal of Type II systems TkoI and TkoII leads to minor transformation efficiency increases, suggesting that the MTase activity of TkoI and TkoII plays a greater role than the REase activity in maintaining genomic stability. Removal of Type IV McrBC systems, which only have REase activity, lead to greater transformation efficiency, suggesting Type IV RM systems and the presence of methylated cytidines on foreign DNA are the major limiting factor in transformation of *T. kodakarensis*.

**Figure 4:**
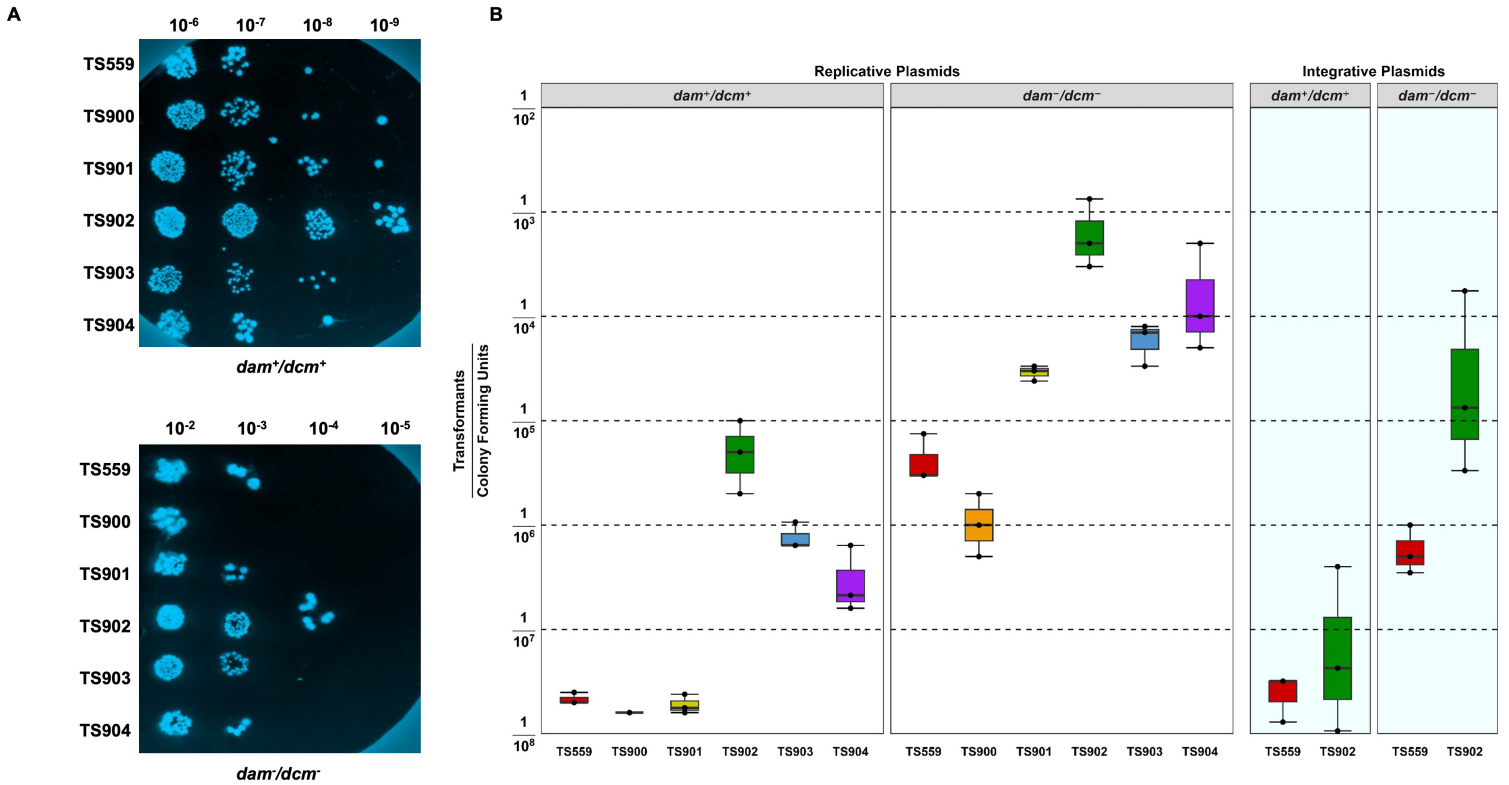
Strains lacking McrBC endonuclease components have drastically increased transformation efficiency **(A)** Representative plates from transformation efficiency assays with spot-diluted parental colonies (top) and spot-diluted transformations with plasmid DNA extracted from *dam*^−^*/dcm*^−^ *E. coli* (bottom) **(B)** Transformation efficiency with autonomous plasmids but not integrative plasmids improved in strains lacking McrBC components. Using unmethylated autonomous or integrative plasmid DNA further increased transformation efficiency in all strains.

The transformation efficiency trends for TS900, TS901, TS902, TS903, and TS904 with plasmids extracted from *dam*^+^*/dcm*^+^ *E. coli* were relatively similar when transforming with plasmids extracted from *dam*^−^*/dcm*^−^ *E. coli*. However, the use of unmodified plasmid DNA resulted in 2-orders of magnitude greater transformation efficiency compared to transformations with unmodified DNA. Transforming TS902 with unmodified pTS543 DNA led to ~1 in 1000 cells being transformed (Figures 4A, B). We then compared the transformation efficiency of TS559 and TS902 with methylated or unmethylated plasmid DNA from a pTS700-based plasmid that must integrate into the genome to confer agmatine prototrophy, TS902 had marginally better transformation efficiency than TS559 with integrative plasmids extracted from *dam*^+^*/dcm*^+^ *E. coli*, while transformations with unmodified plasmid DNA yielded ~1.5-orders of magnitude greater transformation efficiency.

## Discussion

Manipulation of the genome of *T. kodakarensis* originally employed prototrophic-auxotrophic markers to replace or alter genomic loci (Sato et al., 2003, 2005). The dearth of effective prototrophic-auxotrophic selectable markers soon demanded development of antimicrobial resistance markers and selections that could be employed on nutrient-rich media to speed construction of desirable strains (Matsumi et al., 2007; Fukuda et al., 2008; Santangelo et al., 2010; Hileman and Santangelo, 2012; Gehring et al., 2017; Liman et al., 2022). Although effective to generate strains with individual genomic changes, the original modifications often resulted in undesirable retention of selective markers or scars in the genome (Farkas et al., 2013). The implementation of counter-selectable markers that could be recycled to repetitively manipulate different segments of the genome to generate more complex genotypes accelerated progress yet further (Sato et al., 2005; Santangelo et al., 2010). An additional leap of genetic prowess was made possible by construction of autonomously-replicating expression vectors that can be shuttled between *T. kodakarensis* and *E. coli* (Santangelo et al., 2008; Catchpole et al., 2018). Finally, identification and removal of two restriction systems from *T. kodakarensis* increased transformation efficiency by nearly two-orders of magnitude (Zatopek et al., 2021).

The genomes of parental strains have generally increased in complexity to keep pace with the development of increasingly sophisticated genetic selections. Several facile genetic systems have been developed to permit markerless modification of the *T. kodakarensis* genome with base pair resolution to delete sequences, inactivate promoters or gene products, insert sequences that encode new activities, or control expression of any genomic sequences. However, some undesirable remnants of the original genotypes have been retained in many strains, hindering aspects of strain construction. To permit all current genetic selections and plasmids to be utilized without complications from undesirably retained genomic modifications we developed a novel strain of *T. kodakarensis* (termed TS900) whose genome is markerlessly- and cleanly-modified to promote genetic investigation of archaeal physiology. TS900 permits uracil-, mevinolin- (simvastatin), tryptophan- and agmatine-based selections, 6-methylpurine- (6-MP), and 5-fluororatic acid (5-FOA)-based counterselections, and is compatible with all current replicative expression vectors.

The utility of *T. kodakarensis* as a model system for hyperthermophilic archaeal physiology was originally limited by the absence of naturally occurring replicative plasmids. Close-relatives among the *Thermococcales* were identified that did maintain autonomously-replicating small plasmids and a wide-diversity of such plasmids have now been described (Prieur et al., 2004; Santangelo et al., 2007, 2010, 2011; Soler et al., 2007, 2010, 2011; Gonnet et al., 2011; Farkas et al., 2013; Gorlas et al., 2013, 2014; Krupovic et al., 2013; Gehring et al., 2017; Catchpole et al., 2018; Liman et al., 2022). By cloning the entirety of a small plasmid (pTN1) from *Thermococcus nautilus* (Gonnet et al., 2011) into a common *E. coli* plasmid, then adding expression cassettes for genes that could be selected for in *T. kodakarensis*, the first shuttle vector and expression vectors were established for *T. kodakarensis* (Santangelo et al., 2008). Selection of plasmid transformants and maintenance of replicative shuttle vectors is permitted by selection for one or more new or restored activities: PF1848, encoding HMG-CoA reductase from *Pyrococcus furiosus* confers resistance to mevinolin or simvastatin to all strains (Matsumi et al., 2007); TK0254, encoding *trpE* confers tryptophan prototrophy to previously tryptophan auxotrophic strains (Santangelo et al., 2008); TK0149, encoding *pdaD* confers agmatine prototrophy to previously agmatine auxotrophic strains (Santangelo et al., 2010). A second set of compatible plasmid expression vectors were similarly developed from the small plasmid pTP2 from *Thermococcus prieurii* (Catchpole et al., 2018) that share selective markers with pTN1-derived shuttle vectors. The development of compatible expression vectors permits two plasmids, one generated from pTN1 and another from pTP2 to be stably maintained in the same cell (Catchpole et al., 2018).

**Figure 5:**
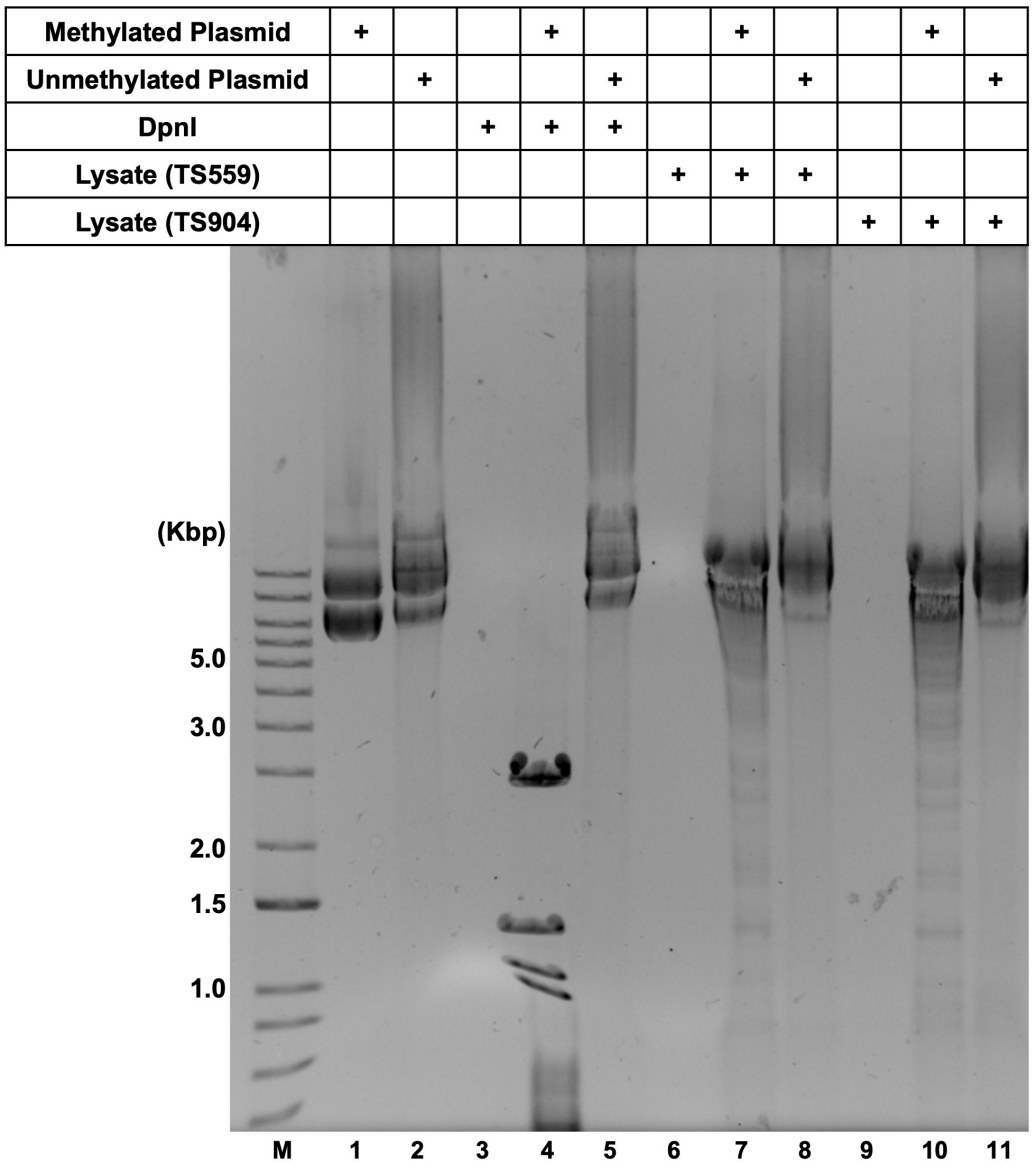
Additional host cell defense systems are present in strain TS904 which lacks all known RM systems. pTS543 DNA extracted from either *dam*^+^*/dcm*^+^ *E. coli or dam*^−^*/dcm*^−^ *E. coli* (lanes 1-2) was treated with either DpnI (lanes 3-5) parental strain TS559 cell lysate (lane 6-8), or TS904 cell lysate (lanes 9-11). pTS543 extracted from *dam*^+^*/dcm*^+^ *E. coli* is heavily degraded when treated with DpnI, TS559 cell lysate, or TS904 cell lysate. pTS543 extracted from *dam*^−^*/dcm*^−^ *E. coli* is unmethylated and therefore unaffected by DpnI, TS559, or TS904 cell lysate.

*T. kodakarensis* retains genetic prowess to continue to serve as model archaeal species for investigation of the totality of extremophile metabolism, molecular biology, and physiology. The continued evolution of parental strains wherein facile but accurate genomic modifications can easily be introduced and rapidly confirmed is essential for continued progress. The development of strains TS900, TS901, TS902, TS903, and TS904 provides new platforms that allow the use of all currently available genetic techniques within a unified strain background. While parental strains typically permit just 1 in ~10^8^ cells to be transformed, here we demonstrate that the simultaneous removal of multiple RM systems and utilization of unmethylated plasmid DNA can increase transformation efficiencies by nearly five orders of magnitude, thereby dramatically increasing the prowess of *T. kodakarensis* for continued genetic investigations into life in the extremes. TS902 demonstrates the greatest transformation efficiency but reduced fitness compared to the parental strain, while TS900 is a unified strain with fitness comparable to TS559, but equally poor transformation efficiency. Each strain has distinct tradeoffs that should be considered prior to use for genetic manipulation. Regardless of which strain is used, transformations with unmethylated plasmid DNAs, integrative or replicative, are ideal for improved plasmid retention during transformations with *T. kodakarensis*. The improvement of transformation efficiency with unmodified plasmid DNA in strains lacking all known RM systems suggests that there may be additional RM systems in *T. kodakarensis* that could be removed to further enhance transformation efficiency. McrBC-like activities may reduce transformation efficiency by degrading exogenous DNA prior to *T. kodakarensis* replication or recombination; supporting the results obtained both with replicative plasmids and integrative plasmids. There is evidence that strongly suggests other systems are operating to cut plasmid DNA that we are now exploring (Figure 5). However, use of strains lacking Type IV RM systems comes at the cost of suboptimal cell growth, suggesting that Type IV RM systems may play extensive roles beyond host defense systems that contribute to cellular fitness in archaea. Although McrBC systems have not been extensively characterized in Thermococcus, homologs are present in related species and share a conserved role of nuclease targeting modified DNA (Hosford et al., 2020; Niu et al., 2020). m^5^C is a very common epigenetic modification in Bacteria and Eukarya, known to improve stability of DNA at high temperatures yet has not been identified in Archaea. While McrBC systems are not characterized to have MTase activity, we performed total deoxynucleoside analysis of all strains to determine if archaeal McrBC modifies genomic DNA and identified a significant absence of m^6^A in strains TS901, TS902, TS903, and TS904 due to the lack of TkoI and TkoII RM systems. The number of m^5^C modifications, however, was under the detection threshold, suggesting McrBC likely contributes to cellular fitness by other means (Rodríguez López et al., 2012; Nardo et al., 2015; Zhao et al., 2022). Deletion of RM systems is not necessary to permit use of the common genetic technologies in *T. kodakarensis*, and while removal of RM systems does not increase the efficiency of transformation with integrative plasmids for genetic modification, the use of unmodified plasmid DNA does assist with transformation efficiency which should accelerate progress in strain constructions.

## Data Availability

The NCBI GenBank accession numbers for TS900-TS904 are, respectively: CP116885.1, CP116886.1, CM161543.1, CM161541.1, and CM161542.1.
