# Supplementary material for: Removal and bypass of restriction modification systems increases transformation efficiency of Thermococcus kodakarensis
Source: Front Microbiol. 2026 Jul 7;17:1855967. doi: 10.3389/fmicb.2026.1855967 (PMC13385129; doi:10.3389/fmicb.2026.1855967)
Supplement: Supplementary file 1 [file Data_Sheet_1.pdf]

Supplementary Material

Removal and bypass of restriction modification systems increases transformation efficiency of *Thermococcus kodakarensis*

Alexander M. Alon[1,2†], Christopher M. Sanders[1†], Brett W. Burkhart[1], Thomas J. Santangelo[1,2*]

[1]Department of Biochemistry and Molecular Biology, Colorado State University, Fort Collins, CO, 80523, USA.

[2]Graduate Program in Cell and Molecular Biology, Colorado State University, Fort Collins, CO, 80523, USA.

*** Correspondence:**
Thomas J. Santangelo
thomas.santangelo@colostate.edu

†These authors contributed equally to this work and share first authorship

1 Supplementary Figures and Tables

1.1 Supplementary Figures

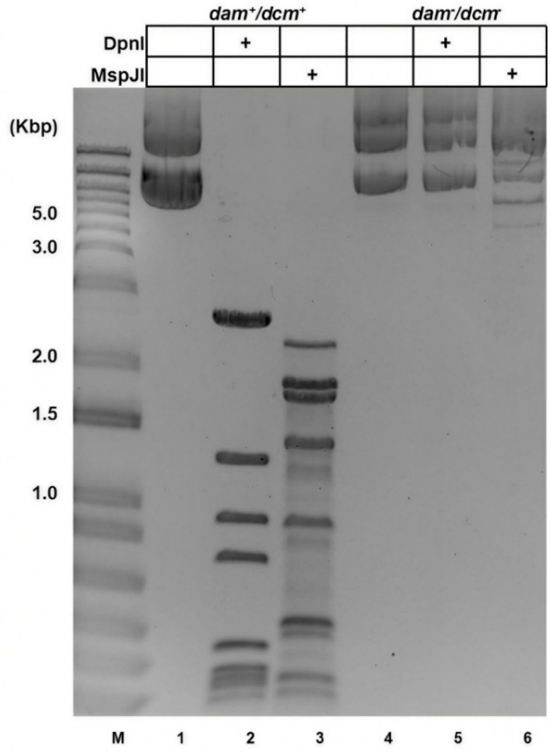

Supplemental Figure 1. Plasmid DNA extracted from *dam⁻/dcm⁻ E. coli* bypasses detection and subsequent degradation by RM systems. pTS543 DNA extracted from either *dam⁺/dcm⁺* or *dam⁻*

/dcm^- *E. coli* was treated with either DpnI (lanes 2 and 5) or MspJI (lanes 3 and 6). pTS543 extracted from *dam*$^+$/*dcm*$^+$ *E. coli* is heavily degraded when treated with either endonuclease (lanes 2 and 3) while pTS543 extracted from *dam*$^-$/*dcm*$^-$ *E. coli* is unmethylated and therefore unaffected by either endonuclease (lanes 5 and 6).

1.2 Supplementary Tables

Supplemental Table 1. Nucleotide sequences of primers A-N used in screening for confirmed deletion of RM systems in TS900, TS901, TS902, TS903, and TS904.

Primer	Sequence 5' - 3'	Locus
A	GGAGGTGAATTTCAACGTTTCCATAACATCGAAGAGC	~700 bp upstream TK0254
B	GGTGAAGGATTTCCTTAACGAACTCAACCATTTCTGC	~700 bp downstream TK0254
C	GGAGGTGAATTTCGTCTCGTCGTGTGCAACTATAACG	~700 bp upstream TK2276
D	GGTGAAGGATTTCAATAACTTCCTCCCACTTCTTCTGG	~700 bp downstream TK2276
E	GGAGGTGAATTTCAATTTCCTCATCGAGTCTCCTTGC	internal primer TK2276
F	GGTGAAGGATTTCAGAGGGGGATAAGGATTTGACTCC	internal primer TK2276
G	GGAGGTGAATTTCGTGATATTCATCGACGAGATTGACG	~700 bp upstream TK1158
H	GGTGAAGGATTTCTCCTTTCTCTAGCACATAATACAGG	~700 bp downstream TK1158
I	GGAGGTGAATTTCGATGAGAAACTGGTGGAGATAAGG	~700 bp upstream TK1460
J	GGTGAAGGATTTCATGAAGTACTTGACTATCTCCTTCG	~700 bp downstream TK1460
K	GACGTCTATCATCTCCTCAAGTGG	~700 bp upstream TK0794
L	CTACTCTACGGCATAAGCAACTACG	~700 bp downstream TK0795
M	GCGTTGAGGTCATTGAGGAGT	~700 bp upstream TK1009
N	CGAACACTTACCTCCTCCAGATGC	~700 bp downstream TK1010

Supplemental Table 2. Predicted product sizes for primer pairs used in screening for presence or absence of RM systems in all strains used in this study.

Primer Pair	Strain	Product Size	Locus
A/B	KOD1	2,537 bp	TK0254
	TS559	2,013 bp	
	TS900	1,235 bp	
C/D	KOD1	1,940 bp	TK2276
	TS559	1,298 bp	
	TS900		
A/F	KOD1	-	-
	TS559	1,394 bp	TK0254
	TS900	-	-
E/B	KOD1	-	-
	TS559	774 bp	TK0254
	TS900	-	-
G/H	KOD1	4,671 bp	TK1158
	TS559		
	TS900		
	ΔTkoIΔTkoII	1,293 bp	
	TS901		
I/J	KOD1	5,120 bp	TK1460
	TS559		
	TS900		
	ΔTkoIΔTkoII	1,271 bp	
	TS901		
K/L	KOD1	4,848 bp	TK0794/TK0795
	TS559		
	TS900		
	ΔTkoIΔTkoII		
	TS901		
	TS902	1,315 bp	
	TS903	4,848 bp	
	TS904	1,315 bp	
M/N	KOD1	5,594 bp	TK1009/TK1010
	TS559		
	TS900		
	ΔTkoIΔTkoII		
	TS901		
	TS902		
	TS903	1,675 bp	
	TS904		
